# Prevalence of hepatitis B and C virus infections among visceral leishmaniasis patients: a systematic review and meta-analysis

**DOI:** 10.3389/fmicb.2024.1415330

**Published:** 2024-06-25

**Authors:** Muluneh Assefa, Sirak Biset

**Affiliations:** Department of Medical Microbiology, School of Biomedical and Laboratory Sciences, College of Medicine and Health Sciences, University of Gondar, Gondar, Ethiopia

**Keywords:** hepatitis B virus, hepatitis C virus, visceral leishmaniasis, systematic review, meta-analysis

## Abstract

**Background:**

Visceral leishmaniasis (VL) patients are at high risk of acquiring hepatitis B virus (HBV) and hepatitis C virus (HCV) infections during multiple injections and the anti-leishmanial treatment possesses a potential hepatotoxic effect. This systematic review and meta-analysis determined the pooled prevalence of HBV and HCV infections in VL patients.

**Methods:**

This study was registered in the International Prospective Register of Systematic Reviews (PROSPERO), with the assigned number CRD42024516889, and conducted as per the Preferred Reporting Items for Systematic Reviews and Meta-Analyses (PRISMA) guidelines. A literature search was performed using PubMed, Medline, EMBASE, Google Scholar, Web of Science, and Science Direct databases. Data were extracted using Microsoft Excel and analyzed using STATA version 11.0 software. A random-effects model was used to estimate the pooled effect size of outcome variables across studies with a 95% confidence interval and was displayed in a forest plot. The *I*^2^ statistic was used to check for heterogeneity. The presence of publication bias was determined using a funnel plot and Egger’s test with a *p* value <0.05 evidence of statistically significant bias.

**Results:**

Among 216 retrieved records, seven studies were eligible for systematic review and meta-analysis. A total of 937 VL patients were examined, revealing that 105 and 93 were infected with HBV and HCV, respectively. The pooled prevalence of HBV was 16.15% (95% CI: −4.10 to 36.39), with a significant heterogeneity (*I*^2^ = 91.4%, *p <* 0.001). The combined prevalence of HCV was 13.74% (95% CI: 1.32–26.16, *I*^2^ = 71.6%, *p* = 0.003). The funnel plot (symmetry), and Egger’s test in both HBV (*p* value = 0.650) and HCV (*p* value = 0.841) revealed no publication bias. In subgroup analysis, high HBV and HCV prevalence was detected in Sudan; 20.64% (95% CI: −13.60 to 54.88) and India; 18.26% (95% CI: −0.40 to 36.92%), respectively.

**Conclusion:**

This study revealed a high prevalence of both HBV and HCV infections in VL patients. In subgroup analysis, the prevalence of HBV and HCV was high in Sudan and India, respectively. Therefore, screening of VL patients for HBV and HCV, vaccination of VL patients in endemic regions, and collaboration between kala-azar and hepatitis elimination programs are required.

**Systematic review registration:**

https://www.crd.york.ac.uk/prospero/export_details_pdf.php#page=1.00&gsr=0, identifier: CRD42024516889.

## Introduction

Leishmaniasis is a neglected vector-borne parasitic disease caused by different species of the *Leishmania* parasite and is endemic in over 90 countries in tropical and subtropical regions worldwide. *Leishmania* is a single-celled parasite that exists in two forms: a motile form (promastigote) found in the vector’s body (female *Phlebotomus* mosquitoes) and a nonmotile form (amastigote) that resides in the host’s body, usually in infected humans or domestic or wild animals (Inceboz, 2019; Sadeghi et al., 2024). A variety of clinical manifestations such as self-resolving skin lesions to life-threatening visceral involvement can be caused by different species of *Leishmania* (Sadeghi et al., 2024). According to the clinical classification, leishmaniasis can be categorized as cutaneous leishmaniasis, mucocutaneous leishmaniasis, visceral leishmaniasis (VL), and post-kala-azar dermal leishmaniasis (Sasidharan and Saudagar, 2021).

Visceral leishmaniasis, commonly known as kala-azar disease is the most severe form that affects the reticuloendothelial system, including the spleen, liver, and bone marrow (Wamai et al., 2020). The etiologic agents of VL varies in geographic location; *Leishmania donovani* in Africa and India, *Leishmania infantum* in the Middle East and Mediterranean regions, and *Leishmania chagasi* in Southern Europe (Scarpini et al., 2022). According to the World Health Organization (WHO) report, an estimated 13,000 cases of VL occurred in 2020 (Ruiz-Postigo et al., 2021). An estimated 500,000 new cases of VL and 50,000 deaths occur annually, which are thought to be underestimated (van Griensven and Diro, 2012).

In nature, humans infected with protozoan parasites can encounter viruses, which could alter their host immune response resulting in exacerbation of pathology and promoting the dissemination of some *Leishmania* infections, based on a hyper-inflammatory reaction driven by type I interferons (Rossi and Fasel, 2018). Because VL treatment consists of intramuscular injections of anti-leishmanial drugs, patients with VL are at a higher risk of contracting dangerous blood-borne infections such as hepatitis B virus (HBV) and hepatitis C virus (HCV). Viral hepatitis is one of the emerging public health problems, which urgently needs special attention (Bhadoria et al., 2022). The global report estimated 295.9 million people were living with chronic HBV infection and 57.8 million people were living with chronic HCV infection. There were more than 3.0 million new infections with HBV and HCV and more than 1.1 million deaths due to the viruses in 2019 (Cui et al., 2023).

The burden of hepatitis viruses in VL patients revealed by a case report showed active HBV and hepatitis D virus co-infection in women with VL complained about low-grade fever, loss of weight, and new onset pancytopenia in known cirrhosis (Lupia et al., 2020). Another report on two cases reported jaundice hepatitis in co-infection of VL and HBV (Coppola et al., 2001; Godoy and de Oliveira Salles, 2002). Additionally, patients with co-infection may be at risk of HBV replication and reactivation, following immunosuppression and acute hepatic necrosis when the immune response resumes (Coppola et al., 2001). A study showed liver damage with significant increases in aspartate aminotransferase, alanine aminotransferase, and total bilirubin, with significantly decreased levels of albumin and platelet count in the co-infection of VL and HBV cases (Adam et al., 2014). Treatment of VL patients with sodium stibogluconate can cause a risk of hepatotoxicity (Mengstie et al., 2021).

Moreover, a case of VL that occurred in a patient with chronic HCV treated with direct-acting antiviral drugs was also described (Colomba et al., 2019). A case report on a Mediterranean VL patient with pre-existing chronic HCV revealed a high plasma concentration followed by a rapidly growing hepatocellular carcinoma suggesting VL causes an increase in HCV replication. The high HCV load was drastically and persistently reduced soon after treatment with liposomal amphotericin B (Precone et al., 2003). The burden of HBV and HCV infections in VL are emerging entity that needs anti-leishmanial treatment modification because most drugs used to treat VL possess a potential hepatotoxic effect. Previously, no data estimated the pooled prevalence of HBV and HCV infections in people infected with VL. Hence, the findings of this systematic review and meta-analysis would help the infection intervention program to hepatitis for minimizing further complications in VL patients.

## Methods

### Study design and protocol registration

This systematic review and meta-analysis was conducted as per the Preferred Reporting Items for Systematic Reviews and Meta-Analyses (PRISMA) guidelines (Moher et al., 2010) (Supplementary Table S1). The protocol has been registered in the International Prospective Register of Systematic Reviews (PROSPERO) under the assigned number CRD42024516889.

### Search strategy

During the literature search, electronic databases such as PubMed, Medline, EMBASE, Google Scholar, Web of Science, and Science Direct were used to retrieve articles reporting HBV and HCV infections in VL patients. A manual search of articles related to the topic was applied and the last search was performed up to February 20, 2024. We used search keywords alone such as “hepatitis B virus,” “HBV,” “hepatitis C virus,” “HCV,” “visceral leishmaniasis,” and “kala-azar” or in combination with Boolean operators such as “OR” or “AND.” The articles retrieved were imported into EndNote X9 bibliographic software manager (Clarivate Analytics, Philadelphia, PA, United States).

### Outcome of interest

The main outcome of interest in this study was the pooled prevalence of HBV and HCV among patients with VL, as described in the original study.

### Studies eligibility

The two authors (MA and SB) screened the titles and abstracts. Full-text articles were then assessed for eligibility, and any disagreements between the authors were resolved through discussion. Articles published regarding HBV and HCV in the English language with a cross-sectional or case–control study design that included the prevalence of HBV and HCV among VL in the results, without limit on country and study period. The studies had to be original research articles and the data had to be presented in a format that allowed for meta-analysis. Case reports, reviews, and meta-analysis studies were excluded from the meta-analysis.

### Risk of bias (quality assessment)

After removing duplicated papers, all potentially eligible papers were reviewed. Full-text papers were retrieved for review and relevant information was extracted. The Joana Briggs Institute (JBI) critical appraisal checklist for simple prevalence was used to assess the quality of included studies (Joanna Briggs Institute, 2017). This tool comprised nine questions. For each question, a score of 0 was assigned for “not reported or not appropriate” and 1 for “yes.” Then, the scores were summarized to obtain a total score ranging from 0 to 9. Based on the assigned points, articles were categorized as having a high (7–9), medium (5–7), or low (0–4) quality. Accordingly, articles with high (7–9) and medium (5–7) quality were included in the final analysis (Supplementary Table S2). Two independent authors (MA and SB) assessed the quality of the studies, and any disagreement was solved by discussion.

### Data extraction

Data were extracted from each study using the designed sheet in Microsoft Excel 2019 (Microsoft Corp., Redmond, WA, United States) by two independent authors (MA and SB). Any ambiguity and difference during extraction were resolved through discussion. The data extracted from eligible studies were author name, publication year, study period, study area/country, study design, number of VL patients, sex (male), age (Mean ± SD or median) number of patients with HBV and HCV infection, and diagnostic method of HBV and HCV.

### Data synthesis

Extracted data were exported to the SATA version 11.0 software for statistical analysis using meta-analysis techniques. A random-effects model was applied to estimate the prevalence of HBV and HCV in VL and 95% confidence intervals were visually displayed using a forest plot. Subgroup analysis was conducted based on the country in which studies reported. The heterogeneity of the included studies was evaluated using an index of heterogeneity (*I*^2^ statistic) value of 0% = no heterogeneity, ≤ 25% = low, 25–50% = moderate, 50–75 = substantial, and ≥ 75% = high (Higgins and Thompson, 2002). A sensitivity analysis to evaluate each study’s impact on the overall pooled prevalence was also performed. Publication bias was statistically investigated using Egger’s test (*p* value <0.05) (Egger et al., 2008) and visual inspection of funnel plots.

## Results

### Search results

**Figure 1:**
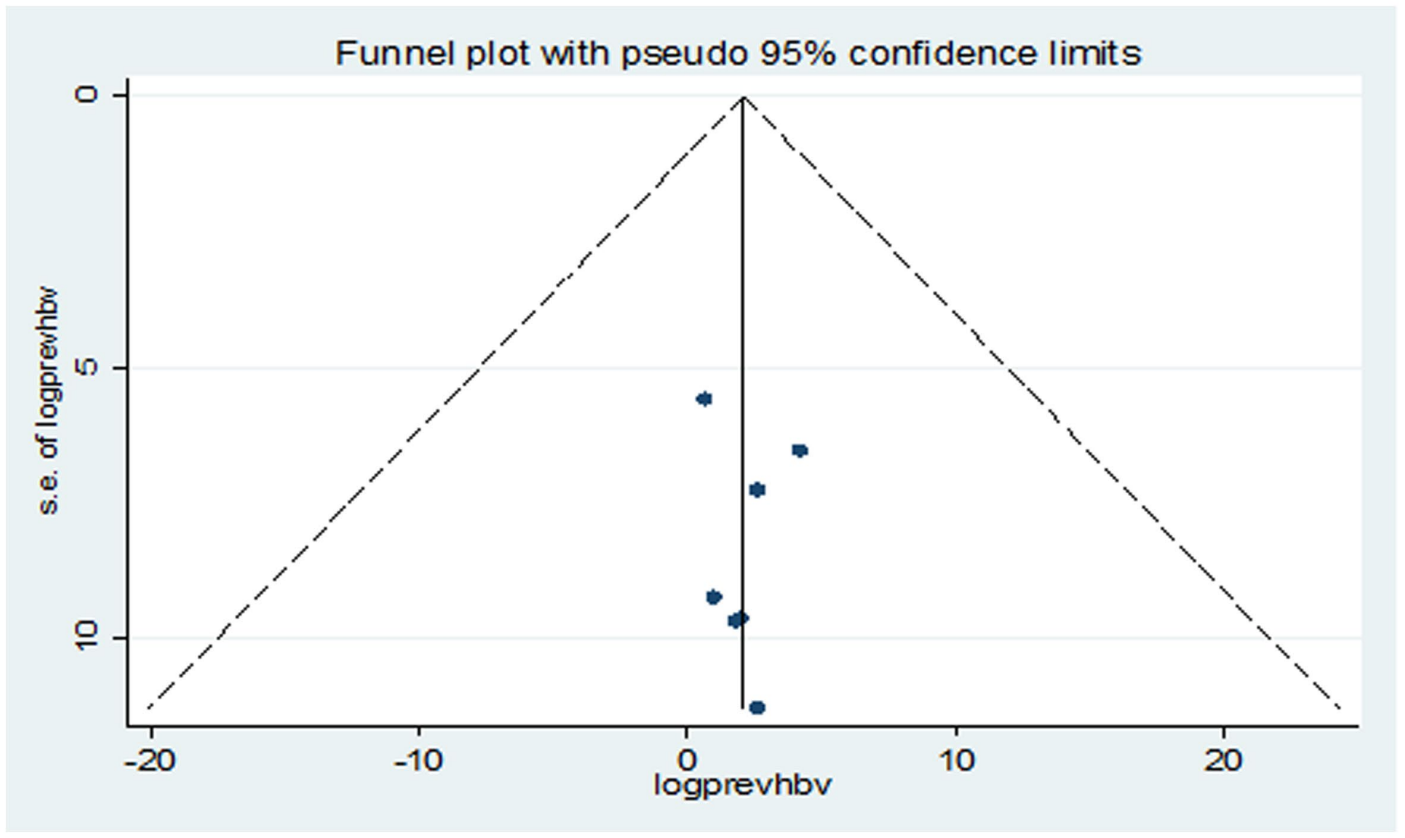
Flow diagram describing the selection of studies for the systematic review and meta-analysis on the prevalence of HBV and HCV in VL patients.

A systematic search retrieved 216 records. After removing records for several reasons (irrelevance of topic, study population, and not published in English language), 110 were screened. Moreover, 43 duplicates were removed and 67 full-text articles were assessed for eligibility. Finally, seven studies were included in the meta-analysis (Figure 1).

### Studies characteristics included in the meta-analysis

In this meta-analysis, a total of seven studies (Singh et al., 2000a,b; Mathur et al., 2008; Adam et al., 2014; Mohammed et al., 2016; Abass et al., 2020; Osman et al., 2023) were analyzed, with a focus on assessing the prevalence of HBV and HCV infection among VL patients. Among these studies, four were conducted in Sudan (Adam et al., 2014; Mohammed et al., 2016; Abass et al., 2020; Osman et al., 2023) while three were conducted in India (Singh et al., 2000a,b; Mathur et al., 2008). Regarding the study design, five studies were cross-sectional (Singh et al., 2000a,b; Mathur et al., 2008; Mohammed et al., 2016; Osman et al., 2023) and two studies were case–control (Adam et al., 2014; Abass et al., 2020). Two studies did not report the study period (Singh et al., 2000b; Abass et al., 2020) and not reported the proportion of male to female ratio (Adam et al., 2014; Abass et al., 2020). The minimum and maximum number of VL patients were 68 (Singh et al., 2000b) and 313 (Mohammed et al., 2016), respectively. Remarkably, a male predominance was observed across the studies. It has been shown that one study (Abass et al., 2020) with zero HCV prevalence (0.0%) had a high effect on pooled effect size and we excluded it from the meta-analysis in case of estimating HCV prevalence. Moreover, a single study reported a 6.4% prevalence of HBV/HCV/VL co-infection (Adam et al., 2014; Table 1).

**Table 1:** Descriptive summary of studies on the prevalence of HBV and HCV infections in VL patients.

Author, year	Country	Study design	Study year	VL patients (*N*)	Sex (M)	Age (Mean ± SD)	Diagnostic method	HBV (*N*)	HCV (*N*)	HBV control (*N*)	HCV control (*N*)	HBV/HCV co-infection
Osman et al. (2023)	Sudan	Cross-sectional	2021	100	71	31.32 ± 12.619	ELISA	7	2	-	-	-
Abass et al. (2020)	Sudan	Case–control	NR	100	NC	≥ 18	ELISA	6	0	5	0	-
Mohammed et al. (2016)	Sudan	Cross-sectional	2013–2014	313	237	31.4 ± 11.9	ELISA	6	4	-	-	-
Adam et al. (2014)	Sudan	Case–control	2008–13	78	NC	NC	ELISA	52	21	-	-	5
Singh et al. (2000a)	India	Cross-sectional	NR	68	53	NC	ELISA	9	14	-	-	-
Singh et al. (2000b)	India	Cross-sectional	1995–1998	164	120	32.5 ± 16.5	ELISA&RIBA-3 test	22	51	7	2	-
Mathur et al. (2008)	India	Cross-sectional	2004–2006	114	96	31 (median)	ELISA	3	1	-	-	-

VL, Visceral leishmaniasis; ELISA, Enzyme-linked immunosorbent assay; RIBA-3 test, Recombinant immunoblot assay; NR, Not reported; NC, Not clear; M, Male; N, Number; SD, Standard deviation.

### Pooled estimates of HBV and HCV infections in VL patients

**Figure 2:**
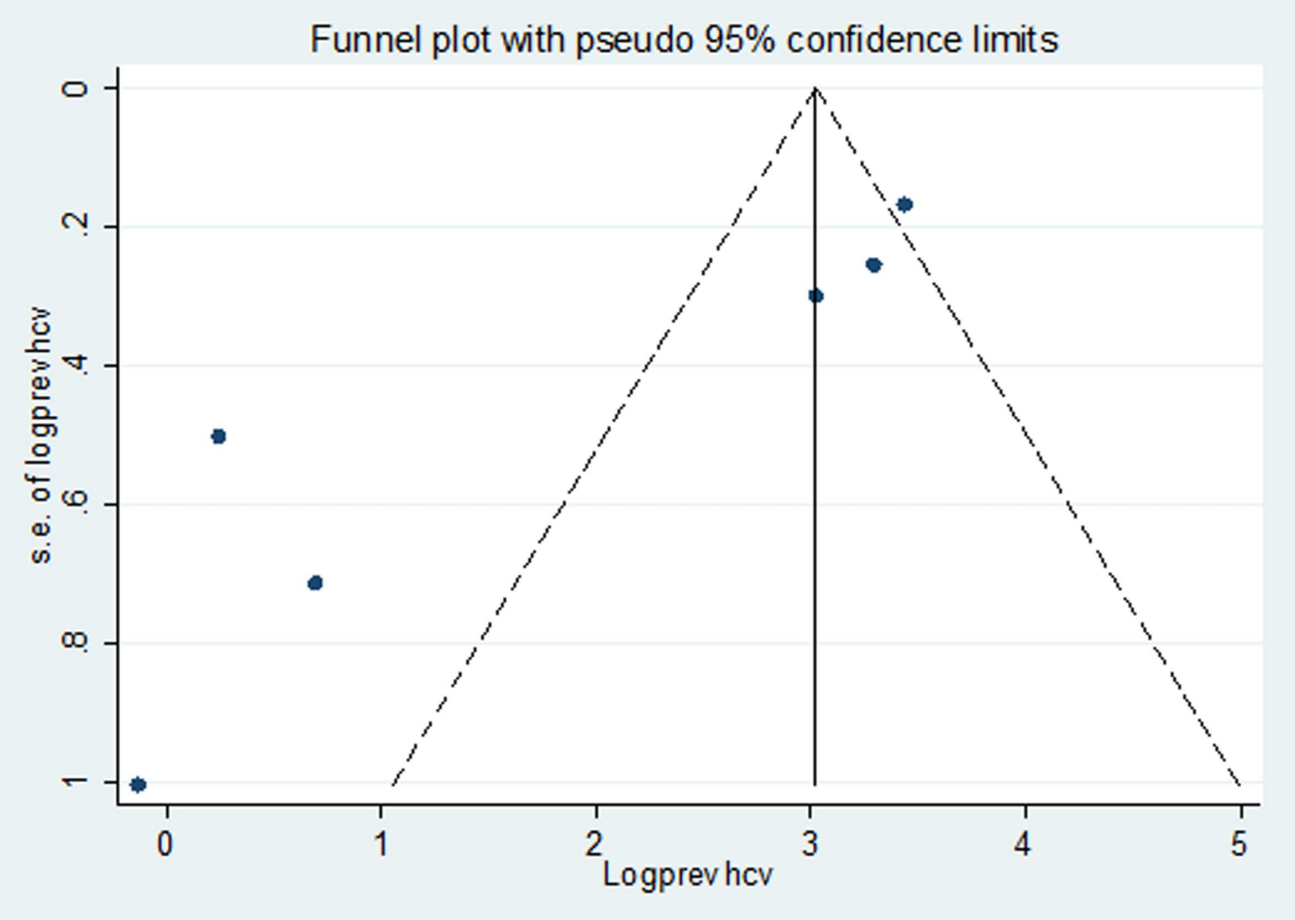
Forest plot showed the pooed prevalence of HBV in VL patients.

**Figure 3:**
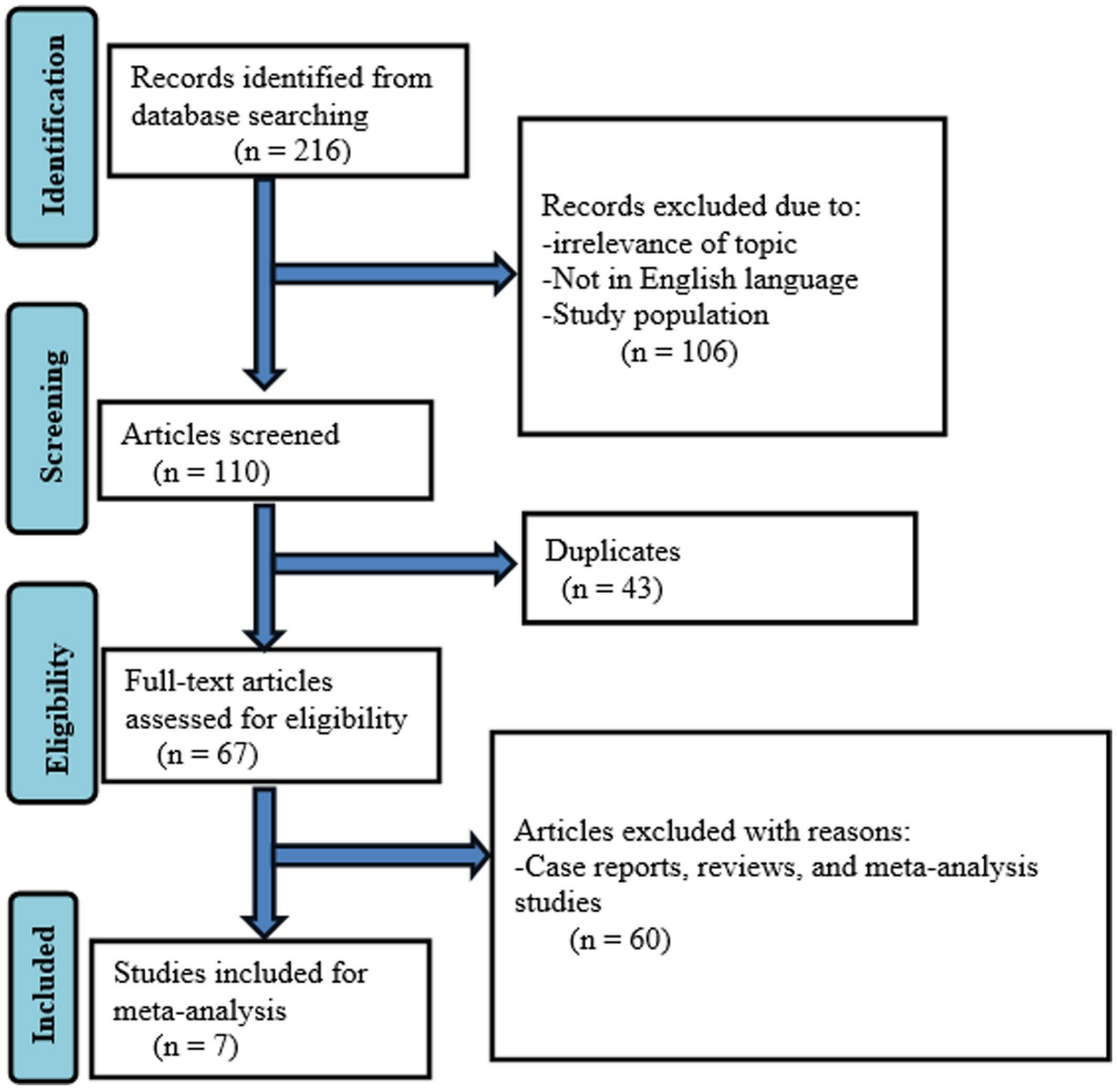
Forest plot showed the HBV prevalence in subgroup analysis based on country.

**Figure 4:**
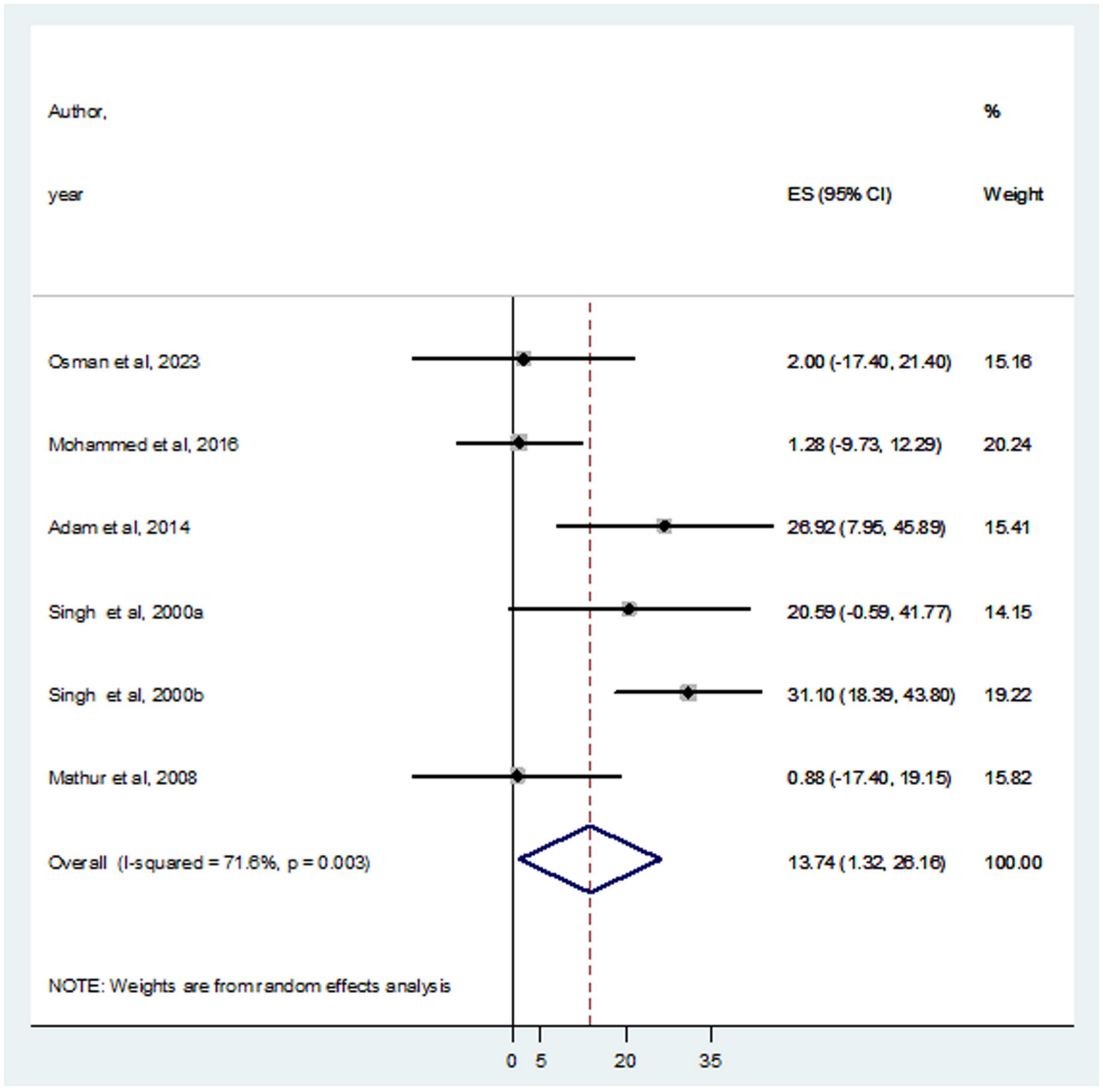
Forest plot showed the pooed prevalence of HCV in VL patients.

**Figure 5:**
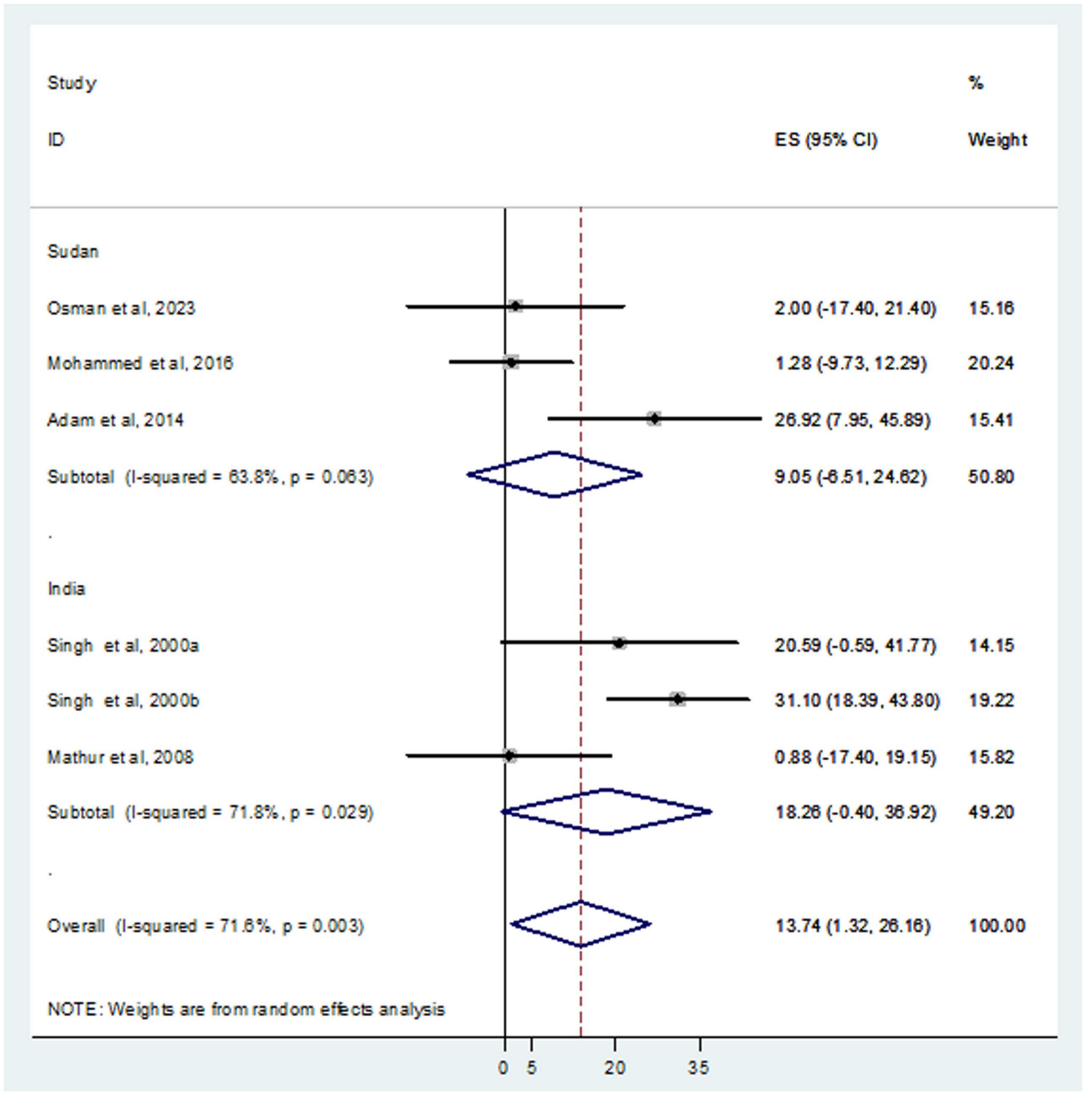
Forest plot showed the HCV prevalence in subgroup analysis based on country.

In this systematic review and meta-analysis, a total of 937 individuals diagnosed with VL were examined, revealing that 105 and 93 were infected with HBV and HCV, respectively. The prevalence of HBV co-infection within individual studies ranged from 1.9% (Mohammed et al., 2016) to 66.7% (Adam et al., 2014). Accordingly, the overall estimated prevalence of HBV infection among patients infected with VL was 16.15% (95% CI: −4.10 to 36.39) (Figure 2). However, significant heterogeneity was found among the included studies (*I*^2^ = 91.4%, *p <* 0.001). Subgroup analysis based on country indicated an HBV prevalence of 20.64% (95% CI: −13.60 to 54.88) in Sudan and 10.10% (95% CI: 0.11–20.09%) in India (Figure 3). On the other hand, the prevalence of HCV infection in the included six studies ranged from 0.9% (Mathur et al., 2008) to 31.1% (Singh et al., 2000a). The combined estimate of HCV infection among patients infected with VL was 13.74% (95% CI: 1.32–26.16, *I*^2^ = 71.6%, *p* = 0.003) (Figure 4). In subgroup analysis, HCV prevalence was 9.05% (95% CI: −6.51 to 24.62) in Sudan and 18.26% (95% CI: −0.40 to 36.92%) in India (Figure 5).

### Publication bias

**Figure 6:**
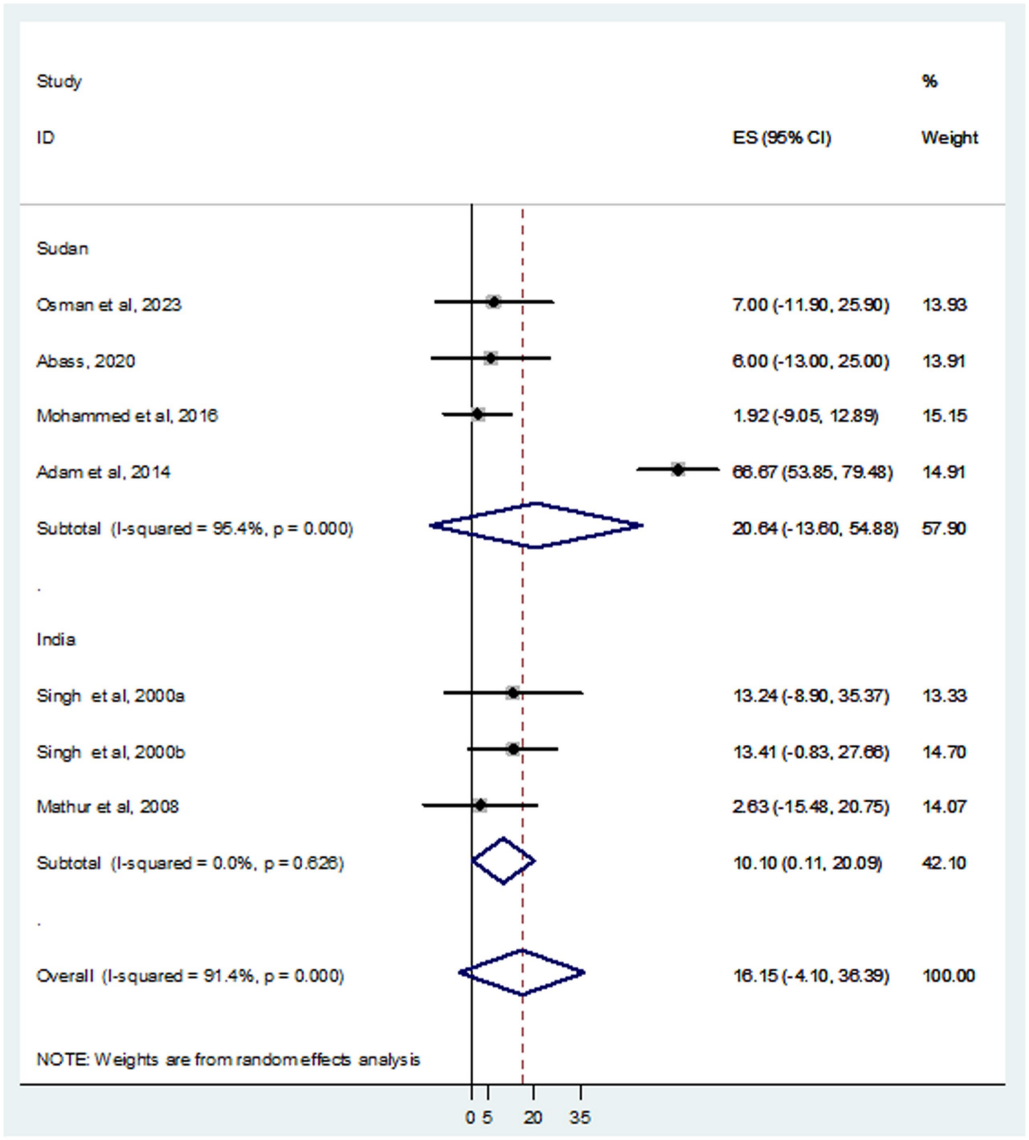
Funnel plot indicated publication bias in the studies on the pooled prevalence of HBV.

**Figure 7:**
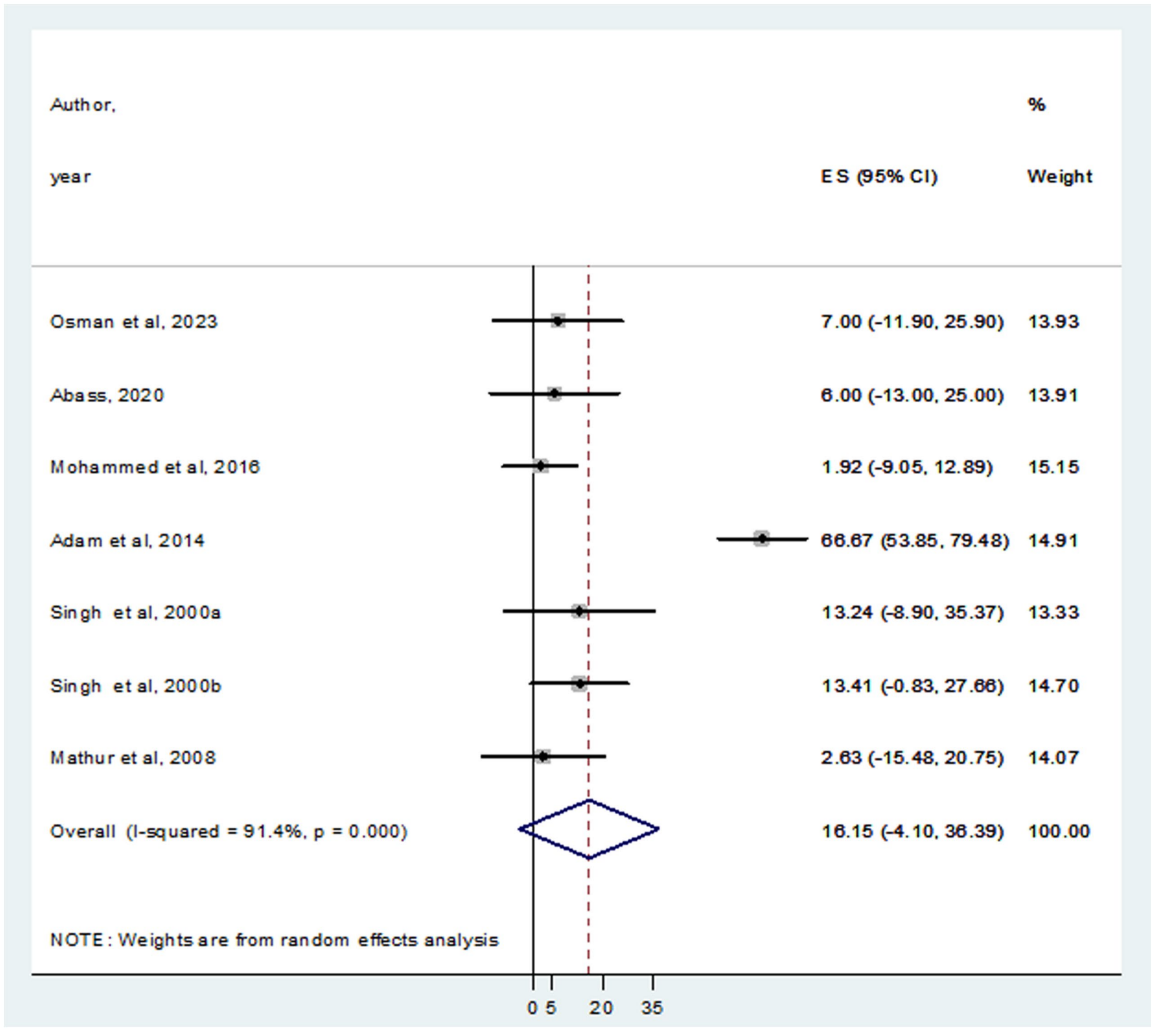
Funnel plot indicated publication bias in the studies on the pooled prevalence of HCV.

Studies were assessed for potential publication bias statistically using Egger’s test and funnel plot. The result of Egger’s test indicated no publication bias in both HBV (*p* value = 0.650) and HCV (*p* value = 0.841) (Tables 2, 3). This was depicted graphically by a funnel plot, which showed a symmetrical display of prevalence reported by the studies (Figures 6, 7).

**Table 2:** Publication bias using Egger test for HBV.

Egger’s test						
Std_Eff	Coef.	Std. Err.	t	*p* > |*t*|	[96% Conf. Interval]	
Slope	40.10906	45. 79126	0.88	0.421	−77.60113	157.8193
Bias	−2.831362	5.874069	−0.48	0.650	−17.93114	12.26841

**Table 3:** Publication bias using Egger test for HCV.

Egger’s test						
Std_Eff	Coef.	Std. Err.	*t*	*p* > |*t*|	[96% Conf. Interval]	
Slope	7.671671	26.82703	0.29	0.789	−66.8121	82.15544
Bias	0.7263741	3.401299	0.21	0.841	−8.717146	10.16989

### Sensitivity analysis

Due to heterogeneity between studies in both HBV and HCV, a sensitivity analysis was performed by step-by-step removal of each study to determine the effect of each study on the pooled prevalence. The results showed that the omitted studies do not have a significant effect on the pooled prevalence of HBV and HCV among VL patients, except the one conducted by Adam et al. (2014), which decreases the pooled estimate of HBV (Tables 4, 5).

**Table 4:** Sensitivity analysis of the included studies to estimate the prevalence of HBV infection among VL patients.

Study omitted	Estimate (95% Confidence interval)	Heterogeneity	
		*I*^2^ (%)	*p* value
Osman et al. (2023)	17.593 (−5.489 to 40.674)	92.7	< 0.001
Abass et al. (2020)	17.754 (−5.266 to 40.775)	92.6	< 0.001
Mohammed et al. (2016)	18.619 (−4.863 to 42.100)	91.3	< 0.001
Adam et al. (2014)	6.416 (−0.053 to 12.885)	0.0	0.834
Singh et al. (2000a)	16.563 (−6.298 to 39.424)	92.8	< 0.001
Singh et al. (2000b)	16.544 (−7.824 to 40.912)	92.8	< 0.001
Mathur et al. (2008)	18.329 (−4.597 to 41.255)	92.5	< 0.001

**Table 5:** Sensitivity analysis of the included studies to estimate the prevalence of HCV infection among VL patients.

Study omitted	Estimate (95% Confidence interval)	Heterogeneity	
		*I^2^* (%)	*p* value
Osman et al. (2023)	15.868 (1.740 to 29.997)	75.3	0.003
Mohammed et al. (2016)	16.994 (3.875 to 30.113)	63.3	0.028
Adam et al. (2014)	11.331 (−2.498 to 25.161)	74.0	0.004
Singh et al. (2000a)	12.594 (−1.707 to 26.896)	76.6	0.002
Singh et al. (2000b)	9.027 (−1.533 to 19.587)	46.9	0.110
Mathur et al. (2008)	16.177 (2.132 to 30.222)	74.4	0.004

## Discussion

The risk of HBV and HCV infections in VL is an emerging problem in hepatic tissue that elevates the liver enzymes such as alanine aminotransferase and aspartate aminotransferase and increases the level of bilirubin (Werneck et al., 2003; Endale et al., 2021). A comparable mechanism to explain the liver involvement in viral hepatitis has been suggested by the recognized relationship between hepatic inflammation and the rise of IL-1β, TNF-α, and IL-6 (Costa et al., 2023). This systematic review and meta-analysis was the first to determine the pooled prevalence of HBV and HCV infections in VL patients.

According to the WHO classification of countries, HBV prevalence >8% can be graded as high, 2–8% is intermediate, and < 2% is considered as low (Evlampidou et al., 2016). In our study, the pooled prevalence of HBV and HCV infections among VL patients was 16.15 and 13.74%, respectively. This indicates the increasing burden of viral hepatitis complicating VL patients that would affect the treatment outcomes resulting in disease severity and mortality. Although there was no previous data about the pooled prevalence of HBV and HCV on VL patients, systematic review and meta-analysis studies reported a combined prevalence of HBV and HCV; 13.7 and 24.7% (Yu et al., 2020), 10.5 and 5.4% (Kenfack-Momo et al., 2022), 4.8 and 1.0% (Wu et al., 2023), 2.66 and 44.82% (Aghaei et al., 2023), 1.97 and 1.88% (Nasiri et al., 2023), and 2.89 and 21.57% (Mehmandoost et al., 2022), respectively. This variation is due to the difference in study populations, hepatitis burden in geographic location, and healthcare provision systems.

Shreds of evidence have described the prevalence of other viral co-infections such as human immunodeficiency virus (HIV). This is described by systematic review and meta-analysis studies conducted on the prevalence estimates of HIV infection among VL patients which reported an overall prevalence of 24.0% in Northwest Ethiopia (Mohebali and Yimam, 2020) and 3.4% in India (Kaur et al., 2024). Another systematic review and meta-analysis on the prognostic factors for mortality of VL patients in East Africa revealed jaundice, HIV, tuberculosis, age > 45 years, edema, bleeding, and hemoglobin ≤6.5 g/dL were strongly associated with mortality (Abongomera et al., 2020). The subgroup analysis based on country showed that the prevalence of HBV among VL patients was higher in Sudan than in India. However, the prevalence of HCV among VL patients was vice versa. This variability may be due to differences in ethnicity, the country’s health provision system, and immune status of the study population, the implementation of an infection prevention system, and adherence to transmission modes of HBV and HCV infections.

Among the studies included in this systematic review and meta-analysis, a single study reported HBV/HCV/VL co-infection (Adam et al., 2014). Studies reported that HBV and HCV co-infection was associated with the sharing of transmission routes, especially in endemic areas and among subjects such as VL patients at risk of parenteral transmissible infections such as blood transfusion and injection drug use (Konstantinou and Deutsch, 2015; Taye et al., 2019). In this study, heterogeneity among studies was high in HBV prevalence, with the lowest and highest prevalence of HBV co-infection in VL infected people was 1.9% (Mohammed et al., 2016) to 66.7% (Adam et al., 2014), respectively. In addition, prevalence of HCV co-infection ranged from 0.9% (Mathur et al., 2008) to 31.1% (Singh et al., 2000a). This disparity could be explained by multiple injections through the use of unsterile needles in health care settings, which is risky for HBV and HCV infections. As described by a study in Sudan, the increased VL and HBV co-infection was related to the predominance of young age study participants, which is partly expected because VL is mainly a disease of children in Sudan (Adam et al., 2014). The VL and HBV co-infection was more prevalent than VL and HCV, reflects the national situation of increased HBV infection.

### Limitations

Although this systematic review and meta-analysis was the first report on the prevalence of HBV and HCV in VL patients, we included only studies in Sudan and India because of the lack of published articles. This suggests that they may not accurately generalize the public health problem worldwide.

## Conclusion and recommendations

This systematic review and meta-analysis demonstrated a high prevalence of both HBV and HCV infections in VL patients. In subgroup analysis, HBV prevalence was high in Sudan whereas HCV was high in Indian VL patients. This requires recognizing the significance of hepatitis and VL co-infection, regular screening of VL patients for HBV and HCV, vaccination of patients, expanding viral hepatitis prevention and control efforts in VL endemic regions, and initiating integrated kala-azar and hepatitis elimination programs.

## Data availability statement

The raw data supporting the conclusions of this article will be made available by the authors, without undue reservation.

## Author contributions

MA: Conceptualization, Data curation, Formal analysis, Methodology, Software, Validation, Writing – original draft, Writing – review & editing. SB: Conceptualization, Data curation, Formal analysis, Methodology, Software, Supervision, Validation, Writing – review & editing.
