# Supplementary material for: Prevalence of hepatitis B and C virus infections among visceral leishmaniasis patients: a systematic review and meta-analysis
Source: Front Microbiol. 2024 Jun 25;15:1415330. doi: 10.3389/fmicb.2024.1415330 (PMC11231734; doi:10.3389/fmicb.2024.1415330)
Supplement: Supplementary file 2 [file Table_2.DOCX]

**Table. S2 Quality assessment of the studies included in systematic review and meta-analysis on the prevalence of HBV and HCV in VL patients.**

| Author, year of publication | Q1 | Q2 | Q3 | Q4 | Q5 | Q6 | Q7 | Q8 | Q9 | Total score (9%) |
| --- | --- | --- | --- | --- | --- | --- | --- | --- | --- | --- |
| Osman et al, 2023 | Y | Y | Y | Y | Y | Y | Y | Y | Y | 9 |
| Abass, 2020 | Y | Y | N | N | Y | Y | Y | Y | Y | 7 |
| Mohammed et al, 2016 | Y | Y | Y | Y | Y | Y | Y | Y | N | 8 |
| Adam et al, 2014 | Y | Y | N | N | Y | Y | Y | Y | Y | 7 |
| Singh et al, 2000a | Y | Y | N | Y | Y | Y | Y | Y | NA | 7 |
| Singh et al, 2000b | N | Y | Y | Y | Y | Y | Y | Y | Y | 8 |
| Mathur et al, 2008 | N | Y | Y | Y | Y | Y | Y | Y | Y | 8 |

**Key:** **Y** = Yes; **N** = Not reported, **NA** = Not appropriate

**Question codes:**

1. Was the sample frame appropriate to address the target population?

2. Were study participants sampled in an appropriate way?

3. Was the sample size adequate?

4. Were the study subjects and the setting described in detail?

5. Was the data analysis conducted with sufficient coverage of the identified sample?

6. Were valid methods used for the identification of the condition?

7. Was the condition measured in a standard, reliable way for all participants?

8. Was there appropriate statistical analysis?

9. Was the response rate adequate, and if not, was the low response rate managed appropriately?
